# Effects of Yoga and naturopathic intervention on cardiac autonomic functions in chronic kidney disease with coronary artery disease – A Case Series

**DOI:** 10.1016/j.jaim.2025.101261

**Published:** 2025-11-07

**Authors:** Deepa Yoganathan, Vimal Vadivel, Vaishali Narayanan, Maheshkumar Kuppusamy, Meenakshi Venkatesan

**Affiliations:** a Department of Manipulative Therapy, Govt. Yoga & Naturopathy Medical College & Hospital, Chennai, India; b Department of Yoga & Naturopathy, Govt. Yoga & Naturopathy Medical College & Hospital, Chennai, India; c Department of Physiology, Govt. Yoga & Naturopathy Medical College & Hospital, Chennai, India

**Keywords:** Chronic kidney disease, Coronary artery disease, Yoga, Naturopathy, Heart rate variability, Parasympathetic nervous system, Complementary medicine

## Abstract

Chronic kidney disease (CKD) and coronary artery disease (CAD) are significant global health challenges with high mortality rates. Conventional treatments often yield limited satisfaction, necessitating exploration of integrative therapeutic approaches. Four patients (3 males, 1 female; ages 39–56) with confirmed CKD (eGFR <15 ml/min) and CAD were enrolled. Patients presented with symptoms including breathing difficulties, chest heaviness, and reduced cardiac function, and were unsatisfied with previous conventional treatments. Patients underwent a 15-day intense program combining yoga, acupuncture, hydrotherapy, manipulative therapy, and specialised naturopathic diet. Daily yoga sessions lasted 60–75 minutes and included specific *asanas*, breathing techniques and relaxation practices. Heart rate variability (HRV) analysis revealed significant improvements across multiple autonomic function markers, observed across all patients. Mean RR intervals increased, with case 3 rising from 582 ms to 675 ms. RMSSD improved from 22.5 ms to 46.9 ms in case 1, indicating enhanced parasympathetic activity. The Stress Index decreased across all cases, most notably in case 3 from 96.4 to 44.8. The LF/HF ratio approached more balanced levels, with case 3 moving from 2.633 to 0.995, suggesting improved autonomic regulation. The integrated yoga and naturopathic intervention demonstrated potential in improving cardiac autonomic functions in CKD and CAD patients. Results suggest these complementary approaches may offer a promising adjunct to conventional medical management by modulating autonomic nervous system activity.

## 1 Introduction

Chronic Kidney Disease (CKD) has now become a leading cause of mortality worldwide, ranking seventh as leading risk factor for mortality worldwide [1]. Currently, about 10 % of world population are affected by kidney related diseases while the incidence rate of CKD has raised up by 88.76 % between the years 1997–2016 [2]. The Global deaths directly caused by CKD is estimated to be around 5–10 million annually, according to the World Health Organisation. CKD also increases the susceptibility for development of comorbidities of the cardiovascular system, thus indirectly adding to the mortality rate. The classification of CKD is based upon the estimated Glomerular Filtration Rate (eGFR), where the eGFR <15ml/min for a minimum period of 3 months is considered as end stage renal disease (ESRD), resulting in kidney failure. CKD is a complex, multifaceted disease and the management includes pharmacological interventions such as administration of antihypertensive drugs like angiotensin-receptor-blockers, angiotensin-converting enzymes inhibitors, combined with procedure of dialysis. Non – pharmacological management includes modifications in lifestyle including changes in dietary patterns with reduced intake of protein, salt, potassium and phosphate [3].

Coronary Artery Disease (CAD), an atherosclerotic disorder with inflammatory background affecting the cardiovascular system, currently one of the leading causes of mortality worldwide accounting for 7 million deaths annually. The Global Burden of Disease reports that about 43 % of cardiovascular disease (CVD) deaths are related to CAD, with increase in estimated deaths from 9 million in 1990 to 19 million in 2010 among the population of the developing countries like India, China, Latin America and thus becoming major health concern in the modern times [4,5]. CVD remains as the leading comorbidity of ESRD, with CAD present over 50 % of the dialysis population that are aged above 65 years. The mortality rate is higher in CKD patients with CAD, with cardiac problem as primary cause of death. Risk factor of CKD patients developing CAD is linear with the decrease in the eGFR [6]. The management of CAD involves invasive procedure of coronary artery bypass grafting and pharmacological intervention consist of administration of anti-inflammatory drugs such as interleukin- 6 receptor blockers and antiplatelet drugs such as protease-activated receptor-1 inhibitor, while non-pharmacological intervention involves lifestyle modifications, dietary changes and moderate exercise [7,8].

Yoga is a traditional practice prevalent from ancient india that focuses to create a balance between a person's body, mind and soul through various practices of *asanas* (postures), *pranayama* (breathing techniques) and *dhyana* (meditation techniques) [9]. The practice of *pranayama*, meditation along with administration of complementary therapies such as acupuncture, hydrotherapeutic techniques and dietary modifications has shown significant changes among CKD patients and CAD patients, resulting in positive effects on sleep quality, regulating the blood pressure, reducing stress markers and thus improving the overall quality of life [10,11].

Although conventional treatments continue to be the foundations of CKD and CAD management, the growing interest in alternate and complementary approaches can offer additional benefits. We understand that the practice of yoga and naturopathic techniques has impact over the cardiovascular and renal system, yet the specific effect of it on the CKD and CAD patients is still underexplored and thus the following case reports compiled as a series aim to address this knowledge gap by examining the impact of short-term, intensive yoga and naturopathy program on patients with established CKD with CAD. We hypothesize that these interventions will lead to improved clinical outcomes, symptom control, and overall well-being in CKD with CAD patients. As a second hypothesis, we suggest that such interventions may bring favourable effects on cardiovascular risk factor modifications and quality of life in these patients.

## 2 Patient information

### 2.1 Case - 1

A 45-year-old male, a known case of CKD with a reduced eGFR (<15ml/min) and increased serum creatinine level (8.6mg/dl) for past 7 months, had an initial symptom of difficulty in breathing when climbing stairs for past 4 months associated with heaviness of the chest. The patient underwent angiogram in a private hospital that revealed narrowing of left anterior descending coronary artery (LAD) with reduced cardiac ejection fraction (40 %). He was diagnosed CAD, underwent conventional medication but was unsatisfied with the results, hence he approached alternate medical care. He visited the outpatient ward of our hospital on May 24, 2024 and got admitted in inpatient ward. Written informed consent was obtained from the participant.

### 2.2 Case - 2

A 39-years-old male, with a known case of diabetes mellitus type-2 for 10 years, had an initial symptom of breathlessness about 7 months ago, went to a private hospital. An ECG was taken revealing mild Myocardial Ischemia and also a blood investigation was done that revealed elevated serum creatinine level (7.37 mg/dl) with reduced eGFR (<15ml/min) that confirmed the diagnosis of CKD. An angiogram later conducted revealed narrowing of the LAD confirming diagnosis of CAD. After 7 months of conventional medicine and not satisfied with the results, approached the outpatient ward of our hospital on 3rd July 2024. He was admitted in the inpatient ward on the same day. Written informed consent was obtained from the participant.

### 2.3 Case - 3

A 56-year-old female, with a history of dermatitis, was a known case of hypertension for the past 2 years, was diagnosed with CKD after a blood investigation done about last year revealed elevated serum creatinine (9.9 mg/dl) and reduced eGFR (<15ml/min) and she was undergoing conventional medicine. Her initial symptom was heaviness over the chest and an ECHO scan done in a private hospital revealed a reduced cardiac ejection fraction (47 %) and the patient underwent angiogram that revealed narrowing of left circumflex artery. She was diagnosed CAD. She was advised for angioplasty and dialysis but the patient dissatisfied with the conventional medicine, approached alternate medicine care and visited the outpatient ward of our hospital on 13 September 2024. She was admitted in inpatient ward on the same day. Written informed consent was obtained from the participant.

### 2.4 Case - 4

A 46-year-old female, with an initial symptom of difficulty in breathing when climbing a flight of stairs for the past 1 year, visited a private hospital. An ECG taken at the private hospital revealed Inferior wall Myocardial Ischemia (MI) and she underwent conventional medicine. Later the patient reported the symptoms of Gradual swelling over both the legs over the past 3 months. On blood investigation at the same private hospital revealed increased levels of serum creatinine (5.6 mg/dl) and reduced eGFR (<15 ml/min) and the patient was diagnosed as CKD. After a total of 1 year of conventional treatment not yielding satisfactory results, the patient sought alternative health care and so she approached the outpatient ward of our hospital on 24 September 2024. She was admitted on the same day in the inpatient ward. Written informed consent was obtained from the participant. A brief timeline about the occurrence of first symptom, diagnosis, beginning of medication and commencement of intervention is mentioned in Table 1.

**Table 1:** Timeline of events.

Events	Case 1	Case 2	Case 3	Case 4
Occurrence of First symptoms	October 2023	July 2023	August 2024	September 2023
Diagnosis of CKD and CAD	January 2024	July 2023	September 2024	June 2024
Use of medications for CKD and CAD	January 2023	August 2023	July 2024	June 2024
Admission and commencement of intervention	May 24, 2024	July 3, 2024	September 3, 2024	September 24, 2024
Discharge from inpatient ward	June 9, 2024	July 19, 2024	September 19, 2024	October 10, 2024

## 3 Clinical findings

On examination, all the patients were conscious, lethargic and well-oriented with a grade-1 pitting pedal oedema over feet and slight puffiness of the face with normal heart sounds and breath sounds on auscultation. White, scaly, dry skin patches was visible all over the body for case 3.

## 4 Assessment

Heart rate variability (HRV) of the patients was recorded with a dual channel MP 160 (BIOPAC) system with ACQKNOWLEDGE 5.0 software before and after the intervention for assessment purposes. Time domain and frequency domain analysis of the recorded data was done using KUBIOS 4.1.1. The time domain HRV variables analysed includes the mean of the intervals between adjacent QRS complexes (mean RR), the square root of the mean of the sum of the squares of differences between adjacent NN intervals (RMSSD), standard deviation of RR intervals (SDNN), Stress index. The frequency domain of HRV variables, the low-frequency (LF) band (0.04–0.15 Hz) and high-frequency (HF) band (0.15–0.4 Hz) in normalised units and LF/HF ratio were analysed.

## 5 Therapeutic interventions

The patients underwent a 15-day intense program of yoga and naturopathic interventions that includes acupuncture, hydrotherapy, manipulative therapy, daily yoga sessions lasting 60–75 mins and were provided with specialised naturopathic diet (mentioned in Table 2) during their stay in inpatient ward. Specific details about the interventions are provided in Table 3.

**Table 2:** Specialised naturopathic diet given to the patients.

Timings	Food Items	Quantity	Servings per Day
7.30 a.m.	A juice made from any one of the following: Bottle gourd, Ash gourd, Snake gourd, Plantain pith.	200 ml	1
9.00 a.m.	A boiled vegetable salad made by mixer of 2/3 of the following: Radish, Carrot, Snake gourd, Chayote.	150 gm	1
	A fruit salad made by mixer of 2/3 of the following: Guava, Papaya, Gooseberry, Pineapple.	100 gm	1
12.00 noon	A juice made from any one of the following: Guava, Papaya, Gooseberry, Pineapple.	200 ml	1
2.00 p.m.	A fruit Salad made by mixer of 2/3 of the following: Guava, Papaya, Gooseberry, Pineapple.	150 gm	1
4.00 p.m.	A juice made from any of the following: Guava, Papaya, Gooseberry, Pineapple.	200 ml	1
7.00 p.m.	A boiled vegetable salad made by mixer of 2/3 of the following: Radish, Carrot, Snake gourd, Chayote.	120 gm	1
	A fruit salad made by mixer of 2/3 of the following: Guava, Papaya, Gooseberry, Pineapple.	200 gm	1

**Table 3:** The details about various intervention given to the patients.

Name of the Therapy	Name of the Specific Treatment	Duration (mins)	Frequency in 15 days
Yoga	• *Vakrasana* (Spinal Twisted pose)	3 mins	15
	• *Ardha Katichakrasana* (Standing side bending pose)	3 mins	15
	• *Katichakrasana* (Waist rotating pose)	3 mins	15
	• Hand stretch breathing	5 mins	15
	• Hands in and out breathing	5 mins	15
	• *Bhujangasana* (Cobra pose)	6 mins	15
	• Sectional Breathing	15 mins	15
	• Deep relaxation technique	20 mins	15
Acupuncture	• Meridian massage – Urinary Bladder meridian	15 mins	15
	• Acupuncture needling with *moxibustion* – *UB-23 (B/L), UB-25 (B/L), CV-14, LIV-3, SP-6, K-3, K-1.*	20 mins	15
	• Auriculotherapy needling – *SHENMEN* POINT, POINT ZERO.	20 mins	15
	• Reflexology – both soles	10 mins	15
Massage	• Partial Massage – Whole Back with lavender oil	20 mins	15
	• Full Body Massage	60 mins	2
Hydrotherapy and Mud therapy	• Renal Pack	20 mins	15
	• Neutral Hip Bath	20 mins	15
	• Ginger Compress	20 mins	15
	• Steam Bath	15 mins	2
	• Full Mud Bath	40 mins	1
	• Plantain Leaf Bath	20 mins	1
Manipulative Therapy	• Seed Therapy (seeds placed at kidney reflex area in the hand for 1 full day, stimulated by pressing at every 60 mins once)	-	15
	• Magnet Therapy – Magnet poles I and V application	20 mins	15
	• Magnetised water consumption	150 ml	15

## 6 Outcomes

Table 4 demonstrates significant improvements in HRV after yoga and naturopathic interventions in patients with CKD and CAD. Notably, the Mean RR increased from 806 ms to 870 ms in case 2, from 582 ms to 675 ms in case 3 and from 656 ms to 711 ms in case 4. RMSSD showed notable gains, increasing from 22.5 ms to 46.9 ms in case 1, from 20.1 ms to 31.1 ms in case 2, and from 1.5 ms to 5.6 ms in Case 3. SDNN also improved, with case 1 rising from 15.4 ms to 32.6 ms and case 3 increasing from 1.3 ms to 6.8 ms. Furthermore, the Stress Index decreased in all cases, particularly in case 3 from 96.4 to 44.8, while the sympatho-vagal balance improved indicated by the decreased LF/HF ratio, especially in case 3, from 2.633 to 0.995.

**Table 4:** Changes in HRV variables after intervention.

Variables	Case 1		Case 2		Case 3		Case 4	
	Pre	Post	Pre	Post	Pre	Post	Pre	Post
Mean RR (ms)	832	801	806	870	582	675	656	711
PNS index	−0.69	−0.19	−0.93	−0.35	−2.63	−2.13	−2.18	−1.34
SNS index	2.45	1.08	1.81	0.65	16.39	7.06	5.82	3.20
RMSSD (ms)	22.5	46.9	20.1	31.1	1.5	5.6	8.1	20.0
SDNN (ms)	15.4	32.6	15.0	23.4	1.3	6.8	9.9	14.0
Stress Index	24.0	14.0	18.6	13.3	96.4	44.8	27.9	23.6
LF (n.u.)	41.77	18.49	39.15	35.58	72.47	49.72	41.74	29.67
HF (n.u.)	58.01	81.46	64.58	67.38	27.52	49.95	28.25	40.33
LF/HF ratio	0.720	0.227	0.544	0.453	2.633	0.995	0.540	0.422

## 7 Discussion

This study aimed to assess the effects of yoga and naturopathic interventions on cardiac autonomic functions in patients with CKD and CAD. The results demonstrate significant improvements in HRV parameters, suggesting a beneficial impact of these interventions on autonomic regulation in this high-risk population. The analysis of HRV markers such as Mean RR, RMSSD, SDNN, and the LF/HF ratio indicate enhanced parasympathetic activity, reduced sympathetic dominance, and overall improved cardiovascular autonomic balance [12].

The observed increase in Mean RR across cases, notably from 582 ms to 675 ms in Case 3, points to an overall increase in the duration of time between heartbeats, reflecting enhanced parasympathetic tone. These findings align with previous studies, which report that interventions targeting relaxation and stress reduction, like yoga, have a positive influence on autonomic nervous system function. The rise in RMSSD, a marker for parasympathetic activity, further supports this, with Case 1 showing a marked increase from 22.5 ms to 46.9 ms. Such improvements suggest that yoga and naturopathic approaches could help in modulating the autonomic dysfunction often seen in CKD and CAD patients, conditions that are characterized by heightened cardiovascular risk [13,14].

Additionally, SDNN, which reflects overall HRV and is a comprehensive marker of both sympathetic and parasympathetic activity, improved significantly, with Case 1 rising from 15.4 ms to 32.6 ms. This suggests an enhancement in the autonomic balance that may contribute to better cardiovascular outcomes. Notably, the Stress Index, which quantifies the degree of sympathetic activity, decreased across all cases, particularly in Case 3 (from 96.4 to 44.8). This reduction indicates a shift toward reduced sympathetic dominance, an important factor in mitigating the risk of cardiovascular events in this vulnerable cohort [15].

The LF/HF ratio, a key marker in assessing the balance between the sympathetic and parasympathetic branches of the autonomic nervous system, also improved in all cases, reflecting the efficacy of the intervention in reducing sympathetic overactivity. In particular, Case 3 exhibited a notable shift from a high LF/HF ratio of 2.633 to a balanced ratio of 0.995, which is indicative of improved autonomic function [16,17].

In line with existing literature, our findings support the notion that integrative approaches, such as yoga and naturopathy, may play a pivotal role in restoring autonomic balance, especially in patients with chronic comorbid conditions such as CKD and CAD. Chronic kidney disease and coronary artery disease are often associated with autonomic dysfunction, which increases the risk of adverse cardiovascular outcomes. By improving HRV, these interventions could potentially help in mitigating this risk, offering a non-invasive, low-cost adjunct to traditional medical treatments [18,19].

While the results are promising, the study has some limitations. The small sample size and the lack of a control group limit the generalizability of the findings. Further research with larger sample sizes and randomized controlled trials is necessary to confirm these results and establish the long-term benefits of yoga and naturopathic interventions in improving autonomic function and cardiovascular outcomes in CKD and CAD patients. Additionally, future studies should investigate the specific mechanisms through which these interventions impact autonomic regulation, possibly exploring biochemical markers of stress and inflammation.

In conclusion, the findings of this study suggest that yoga and naturopathic interventions are effective in improving HRV and autonomic function in patients with chronic kidney disease and coronary artery disease. These results underscore the potential of integrative therapies as complementary treatments in managing cardiovascular risk in this high-risk population. Further research is warranted to fully understand the underlying mechanisms and confirm the clinical benefits of these interventions.

## 8 Patient perspective

All the four patients were satisfied on their improvement in breathing and felt relaxed, happy and calm at the end of each therapy session. On September 18, 2024, Case 3 reported “All my skin lesions are starting to disappear and I feel confident again over myself”.

## Declaration of patients consent

The authors certify that they have obtained all the appropriate patient consent form. In the forms, patients have given their consent for their clinical information to be reported in the journal. Patients understand that their names, initials and personal details will not be published and due efforts will be made to conceal their identity, but anonymity cannot be guaranteed.

## Author contributions

DY – conceptualization, methodology, investigation, writing-review and editing, supervision. VV – methodology, formal analysis, investigation, data curation, writing-original draft and editing. VN – methodology, investigation, data curation, writing-original draft and editing. MK – validation, investigation, writing-review and editing, supervision. MV – data curation, writing-original draft.

## Funding sources

Nil.

## Conflict of interest

The authors declare that they have no known competing financial interests or personal relationships that could have appeared to influence the work reported in this paper.

## Declaration of generative AI in scientific writing

None

## Data Availability

The datasets generated during and/or analysed during the current study are available from the corresponding author on reasonable request.
